# Finite element analysis of intraosseous distal radioulnar joint prosthesis

**DOI:** 10.1186/s12891-022-05746-3

**Published:** 2022-08-17

**Authors:** Farzaneh Gholamian, Mehran Ashrafi, Ali Moradi

**Affiliations:** 1 Orthopedics Research Center, Mashhad University of Medical Sciences, Mashhad, Iran; 2 Faculty of Biomedical Engineering, Sahand University of Technology, Sahand New Town, Tabriz, Iran

**Keywords:** Distal radioulnar prosthesis, Biomechanics study, Kapandji procedure, Finite element analysis

## Abstract

**Background:**

Joint replacement is one of the options to retrieve the interosseous distal radioulnar joint (DRUJ) function. DRUJ prosthesis has recently been introduced clinically to treat DRUJ instability. This article analyzes the biomechanical behavior of the prosthesis during different loadings by the finite element method.

**Methods:**

CT images of a healthy 33 years old man were used to construct the three-dimensional geometry of the forearm bone. Then two models, a healthy foreman (Model A) and a damaged model with an inserted interosseous prosthesis (Model B), were constructed to analyze and compare the foreman's biomechanical behavior under different loading conditions using the finite element method. Both models were examined during pronation and supination with 500, 1000, 2000, and 5000 N.mm values. Also, both models were subjected to volar and dorsal loads with values of 10, 30, and 50 N and traction force with 100, 150, and 200 N.

**Results:**

Maximum and minimum principal stresses were evaluated for bones in all conditions, and von Mises stress was considered for the prosthesis and fixing screws. In supination, the maximum stress in Model A is significantly higher than the Model B. However, the maximum principal stress of both models is similar during volar and dorsal loading. In Model A, the maximum principal stress in traction is much smaller than in Model B. The absolute value of minimum principal stress in pronation and supination in Model B is higher than in Model A. The prostheses and screws are subjected to higher stresses during pronation than supination. Also, the amount of stress created in prostheses and screws during volar and dorsal loading is almost equal. In traction loading, screws are subjected to significantly high stresses.

**Conclusion:**

Our study indicates that the interosseous DRUJ prosthesis can perform the foreman's normal daily activities. This prosthesis provides the ability similar to a normal hand.

**Level of evidence:**

IV.

## Introduction

The distal radioulnar joint (DRUJ) is a complex joint involved in both forearm axial and wrist movements and the transmission of forces across the wrist to the forearm [1]. Disorders of the distal DRUJ, such as osteoarthritis, ulna fractures, inflammatory arthritis, ligament injuries, and congenital diseases, are associated with wrist ulnar side pain, weakness, instability, and loss of forearm rotation [2, 3].

Different treatment methods have been proposed to treat osteoarthritis and DRUJ instabilities [4–7]. One of the options to retrieve the DRUJ function is joint replacement [8, 9]. Joint replacement in this area is challenging due to its biomechanical complexity. However, studies with long-term follow-up to evaluate the effect of these techniques showed that they cannot restore normal anatomy and kinematics and have not been entirely successful in reducing forearm pain and instability [10, 11].

So far, various designs for distal radioulnar prostheses have been introduced [12–14]. Recently a new category of DRUJ prosthesis by the name of "interosseous DRUJ prosthesis" has been introduced clinically based on the "Kapandji technique" [15]. In the Kapandji technique, a joint is created in the distal region of ​​the ulna by removing a segment of the bone [9]. Moradi et al. [16] performed a study on a cadaver to evaluate the function of DRUJ prosthesis in the body. Based on the results of that study, the intraosseous DRUJ prosthesis did not significantly affect the wrist range of motions and showed efficacy in restoring function. However, unlike significant prosthesis stability in longitudinal traction, the rotational force has inherent instability. In another short-term clinical study, one of the five patients with the DRUJ prosthesis was dislocated in a two-year follow-up [15].

However, two fundamental questions are still unclear about this prosthesis: Since the prosthesis is located in its anatomic place, it may affect the wrist and axial forearm range of motion and concerns about the stability of the prosthesis. According to two previous articles, the latter issue is more concerned. Therefore, using the finite element method, this prosthesis and forearm bone's biomechanical behavior under different loading conditions were evaluated in long-term and compared with a healthy forearm.

## Materials and methods

CT images of a healthy 33 years old man were used to compare the biomechanical behavior of the healthy forearm (model A) and the designed prosthesis (model B) under different loading conditions using the finite element method.

### Geometry

**Fig. 1:**
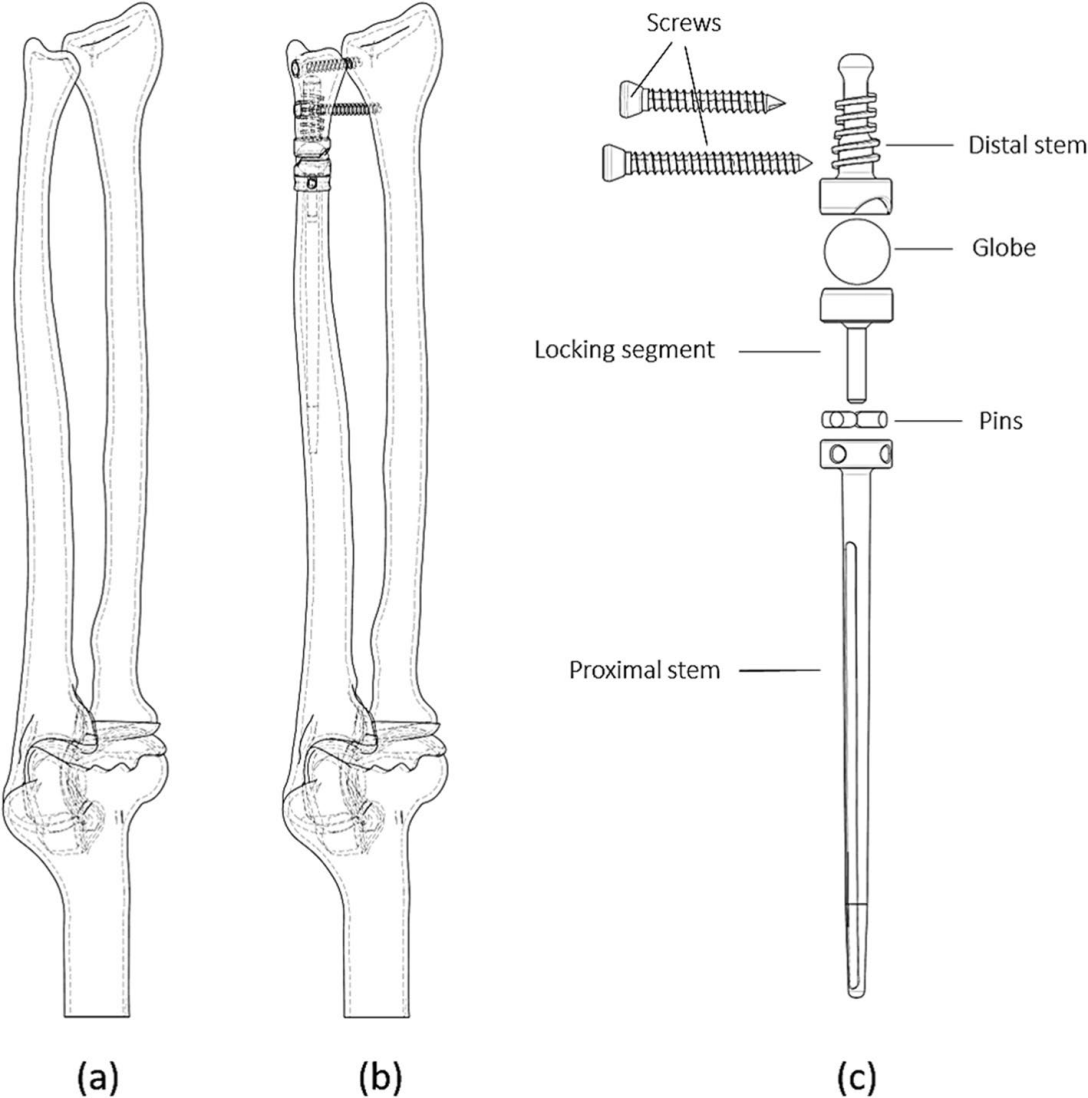
3D representation of wrist and DRUJ prosthesis and their components: (**a**) healthy forearm, (**b**) defeated ulna with the prosthesis, and (**c**) DRUJ prosthesis and its components

**Fig. 2:**
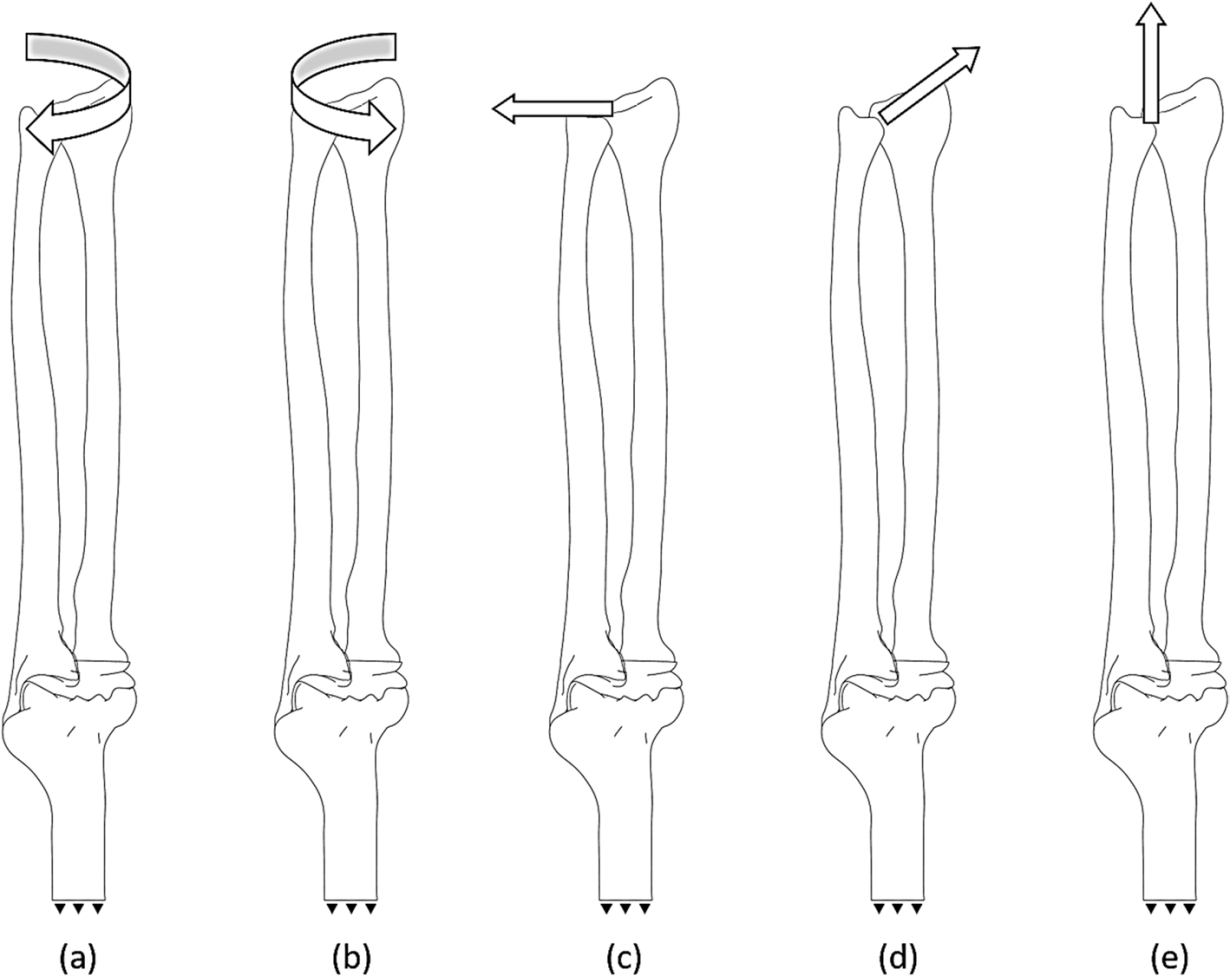
Five loading condition: (**a**) pronation (500, 1000, 2000, and 5000 N.mm), (**b**) supination (500, 1000, 2000, and 5000 N.mm), (**c**) dorsal (10, 30, and 50 N), (**d**) volar (10, 30, and 50 N), (**e**) traction (100, 150, and 200 N)

CT images were used to construct 3D geometry, including ulnar, radius, and distal humerus. After receiving the CT images, MIMICS software (MIMICS 10.1; Materialise NV, Leuven, Belgium) was used to convert the images into STL format, which were finally imported to CATIA (CATIA V5; Dassault Systemes, Ve' Lizy-Villacoublay, France) to construct the final models. Finally, cortical and trabecular bones were modeled by considering a 2 mm layer for cortical bone. Since the CT imaging cannot detect the cartilages, the gap between the bones is considered for modeling the elbow joint cartilages. Next, each cartilage was modeled by expanding the related bone volume and operating Boolean operations with some geometrical modifications. And finally, the humerus was fixed in all directions, as depicted in Fig. 2. Following segmentation in Mimics software, threshold definition for different model components, and creating STL files, then these STL files were transferred to Catia software. Then the defects were corrected in the affected areas. The final CAD files were imported into ABAQUS software (ABAQUS 6.11, Dassault Systèmes, Vélizy-Villacoublay, France). The subsequent finite element procedures are illustrated in the following sections. The final geometry of the model is depicted in Fig. 1.

A specific defect by subtracting a region from the ulna was created to insert the DRUJ prosthesis, as depicted in Fig. 1. The DRUJ prosthesis is inserted between two sides of the osteotomy. Pseudoarthrosis is carried out in the Sauvé-Kapandji procedure to address the drawbacks of different surgical techniques and prostheses [9]. In this prosthesis, two main distal and proximal parts are connected with a ball. This ball enables the prosthesis to move freely in each degree of freedom. Furthermore, the proximal part fixes to ulna and radius with two screws. Finally, the distal and proximal stems centered on the sphere can have axial movements and bending.

### Material definition

All materials used for cortical and trabecular bone, cartilages, and different parts of the prosthesis are considered linear elastic [17]. Also, titanium material properties are assigned to all prosthesis parts except the ball. For the ball, polyurethane [18] linear elastic properties are assigned [15]. The material properties of the bone were defined using Young’s modulus of 17.5 GPa for the cortical bone and 309.8 MPa for the trabecular bone. The Poisson’s ratio for both the cortical and trabecular bones was 0.3. Cartilage was modeled with Young’s modulus of 12 MPa and Poisson’s ratio of 0.4 [17]. Titanium and Polyurethane were assumed to have Young’s modulus of 110 GPa, and Poisson’s ratio of 0.35 and 0.31, respectively [18, 19].

Ligaments stand for tension force between bones modeled with spring elements. Springs were designated to each elbow ligament to model the corresponding ligament's function. Four parallel springs for each medial anterior, medial posterior, lateral radial, and lateral ulnar ligaments with the stiffness of 72.3, 52.2, 15.5, and 57 N/mm were selected, respectively. Three similar springs with stiffness of 28.5 N/mm were assigned for the annular ligament. Also, for each distal/proximal interosseous membrane and central interosseous membrane, two springs with the stiffness of 18.9 N/mm and 65 N/mm were assigned, respectively. The position of these ligaments was selected based on previous studies [17, 20].

### Loading and boundary condition

Cortical and trabecular bones bounded together [21]. As this study aims to analyze the prosthesis's long-term behavior, the interface of the distal and proximal part of the prosthesis is bonded to the bone, and frictionless contact is defined between the ball and the prosthesis. The bones and ligaments of the wrist were not considered in this study, so the ulna and radius’ proximal surfaces connected with coupling constraints [17]. Each ligament bonded to its bone, and the frictionless surface-to-surface contact was considered for the cartilages of humerus-radius, humerus-ulna, and radius-ulna.

Five different loading scenarios were considered for both models A and B. These loading conditions represent different conditions each hand encounters during regular daily activity. These loading conditions include pronation (500, 1000, 2000, and 5000 N.mm), supination (500, 1000, 2000, and 5000 N.mm), dorsal (10, 30, and 50 N), volar (10, 30, and 50 N), and traction (100, 150, and 200 N). 180º rotation was applied to the ulna and radius with the origin depicted in Fig. 2 to simulate the supination loading; then, the corresponding torque was applied. In all simulations, the proximal surface of the humerus was fixed. All loading conditions are depicted in Fig. 2.

### Finite element analysis

ABAQUS-CAE was used to build the finite element meshes with 4-noded linear tetrahedrons. The optimal number of elements was chosen after simulating the convergence analysis to obtain sufficient accuracy in the results, then all simulations were performed using ABAQUS (ABAQUS 6.11, Dassault Systèmes, Vélizy-Villacoublay, France).

## Results

The following results show the stress and displacement distribution of the bone in a healthy forearm (model A), a forearm with a prosthesis (model B), and the prosthesis and screws in different loading conditions.

**Fig. 3:**
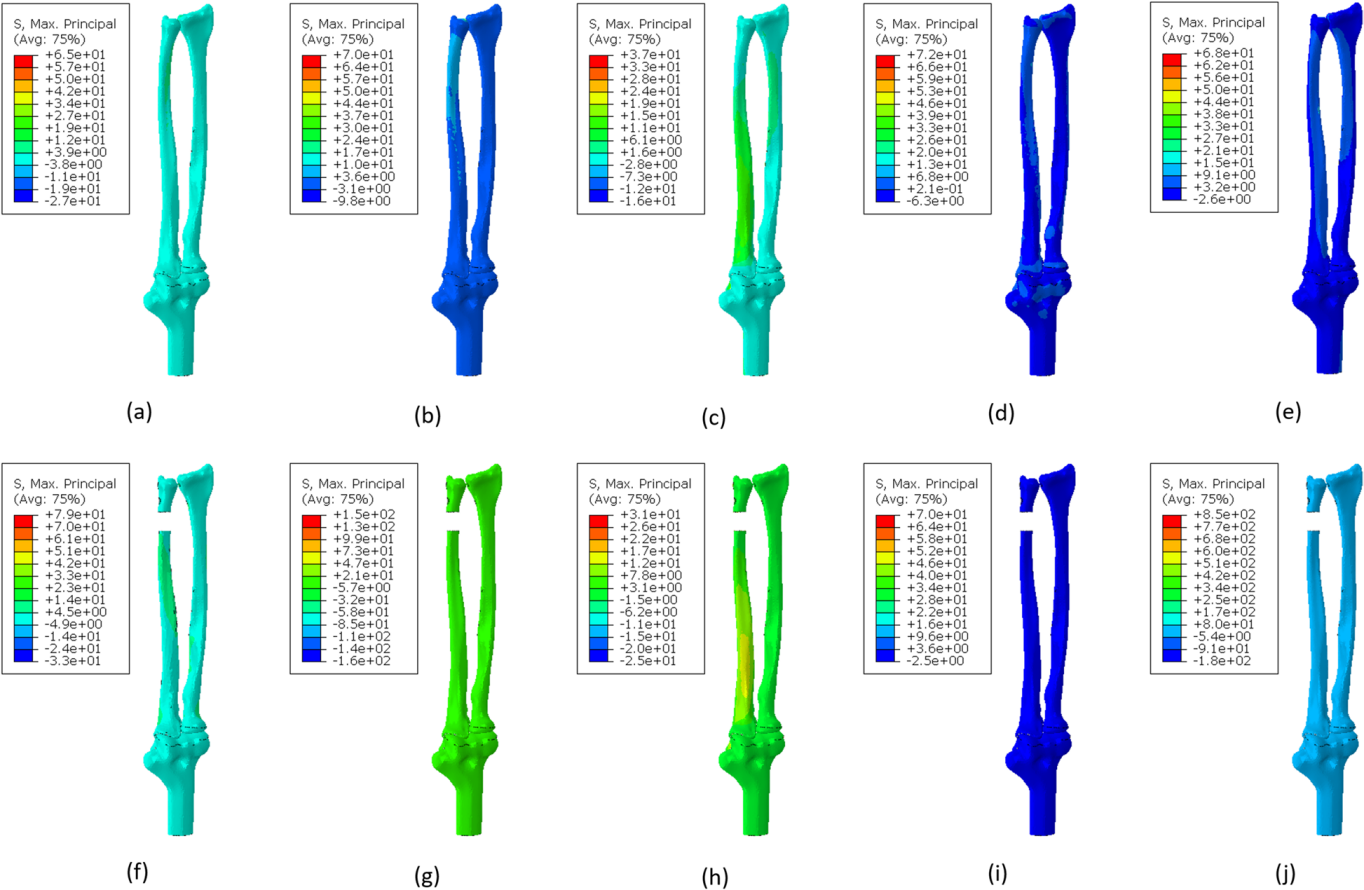
Distribution of maximum principal stress in different loading conditions for model A: (**a**) 1000 N.mm pronation (maximum in the head of the ulna, minimum in the head of the radius) (**b**) 1000 N.mm supination (maximum and minimum on the inner surface of the radius head) (**c**) 30 N volar (maximum and minimum at the end of the ulna bone) (**d**) 30 N dorsal (maximum at the end of the ulna bone and the minimum at the inner surface of the head of the radius bone) (**e**) 150 N traction (maximum in the head of the radius and minimum in the inner surface of the humerus bone), and model B: (**f**) 1000 N.mm pronation (maximum in the middle of the inner surface of the ulna bone and minimum at the outer surface of the head of the ulna) (**g**) 1000 N.mm supination (maximum and minimum in the middle of the external surface of the ulna) (**h**) 30 N volar (maximum and minimum in the middle of the posterior surface of the ulna) (**i**) 30 N dorsal (maximum in the middle of the outer surface of the ulna and minimum at the end of the ulna bone) (**j**) 150 N traction (maximum at the head of the ulna and minimum at the middle of the external surface of the ulna)

Figure 3 shows models A and B's maximum principal stress distribution with different loading conditions. The results of pronation and supination with 1000 N.mm, volar and dorsal with 30 N and traction with 150 N are depicted in this figure. In supination, the maximum stress in model A is significantly higher than the model B. Except for the first loading (5000 N.mm), the maximum principal stress created in models A and B during supination, is almost identical to the maximum principal stress generated during pronation. For example, the maximum principal stress during supination under 2000 N.mm loading is equal to 294 MPa in model B, and the equivalent loading in model B pronation is 273 MPa. Similarly, in model A, the maximum principal stress value during supination was 140.8 MPa under 2000 N.mm loading, equal to 128.9 MPa in model A pronation. It can be seen that the maximum principal stress during supination is slightly higher than pronation.

**Fig. 4:**
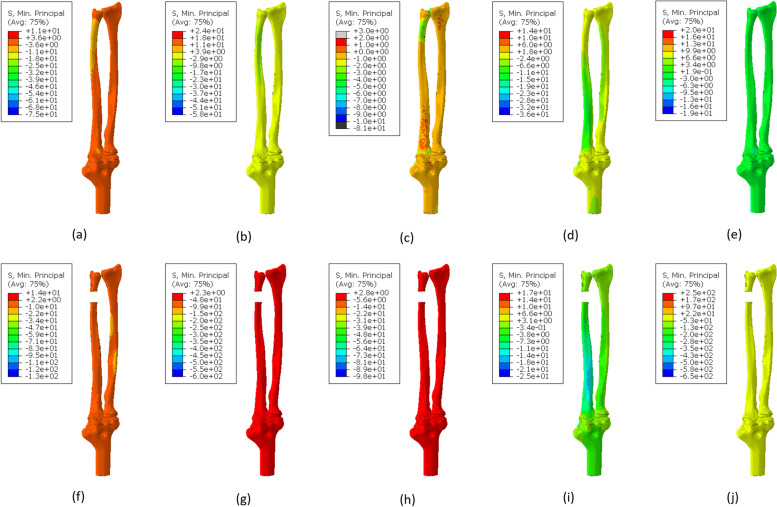
Distribution of minimum principal stress in different loading conditions for model A: (**a**) 1000 N.mm pronation (maximum and minimum in the head of the radius bone), (**b**) 1000 N.mm supination (maximum in the head of the ulna bone and minimum in the head of the radius bone), (**c**) 30 N volar (maximum and minimum at the end of the ulna), (**d**) 30 N dorsal (maximum and minimum at the end of the ulna), (**e**) 150 N traction (maximum in the head of the ulna bone and minimum in the head of the radius bone), and model B: (**f**) 1000 N.mm pronation (maximum on the external surface of the head of the ulna and minimum on the internal surface of the head of the radius bone), (**g**) 1000 N.mm supination (maximum in the middle of the external surface of the ulna and minimum at the head of the ulna), (**h**) 30 N volar (maximum in the middle of the posterior surface of the ulna and minimum at the end of the ulna), (**i**) 30 N dorsal (maximum and minimum in the middle of the external surface of the ulna), (**j**) 150 N traction (maximum in the middle of the posterior surface of the ulna and minimum at the head of the ulna)

The maximum principal stress of both models is similar during volar and dorsal loading. However, the amount of stress during the dorsal is about 70 MPa and in the volar is about 31 MPa. There is a significant difference between model A and model B in traction. In this loading condition, the maximum principal stress in model A is about 68 MPa, while in model B, it is about 850 MPa. Figure 4 shows the distribution of minimum principal stress in each loading condition as depicted for maximum principal stress. In model B the absolute value of minimum principal stress is in pronation, and supination is higher than in model A.

The absolute value of the minimum principal stress in pronation and supination in model B is higher than model A. Still, this difference is more significant in supination than in pronation. For example, under a load of 1000 N.mm, the minimum absolute value of the principal stress in model B during supination is equal to 603 MPa, which is equivalent to this loading in model A, in which a value of 57.71 MPa was obtained. But in pronation, the minimum absolute value of the principal stress during the loading of 1000 N.mm in model B was equal to 132.1, while in model A, the value of 74.92 MPa was obtained.

**Fig. 5:**
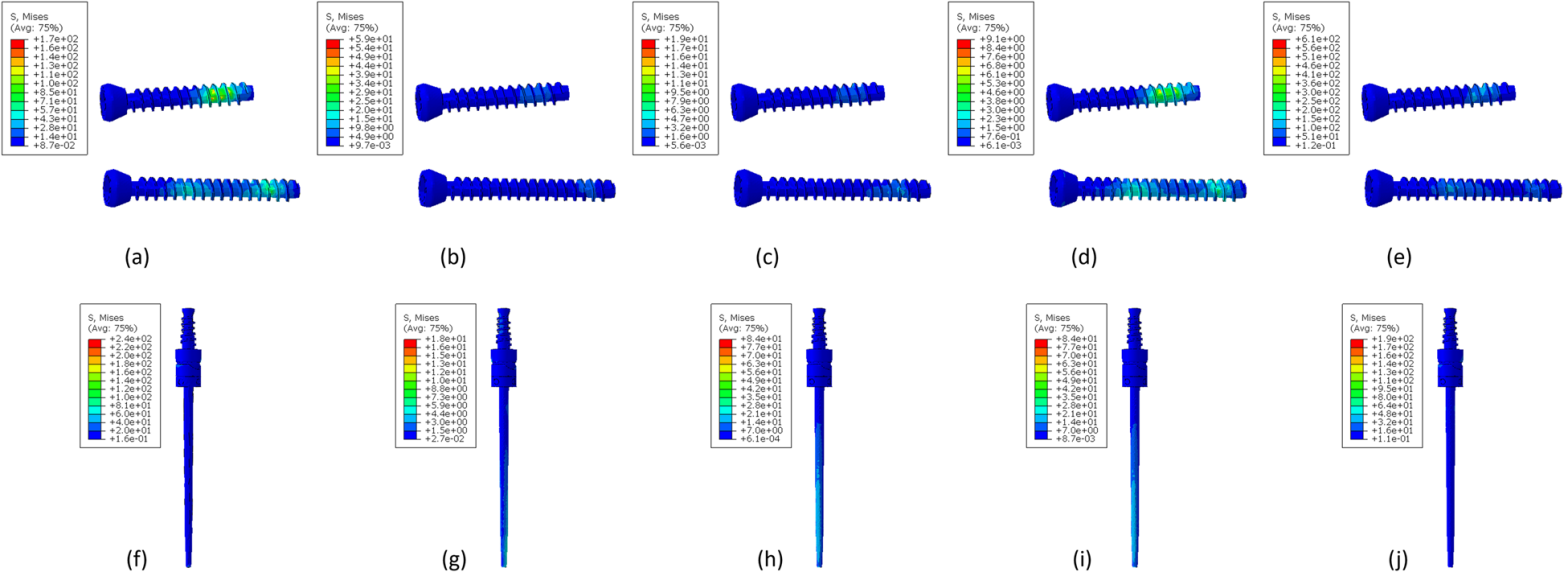
Distribution of von Mises stress in different loading conditions for screws: (**a**) 1000 N.mm pronation, (**b**) 1000 N.mm supination, (**c**) 30 N volar, (**d**) 30 N dorsal, (**e**) 150 N traction, and prosthesis: (**f**) 1000 N.mm pronation, (**g**) 1000 N.mm supination, (**h**) 30 N volar, (**i**) 30 N dorsal, (**j**) 150 N traction

**Fig. 6:**
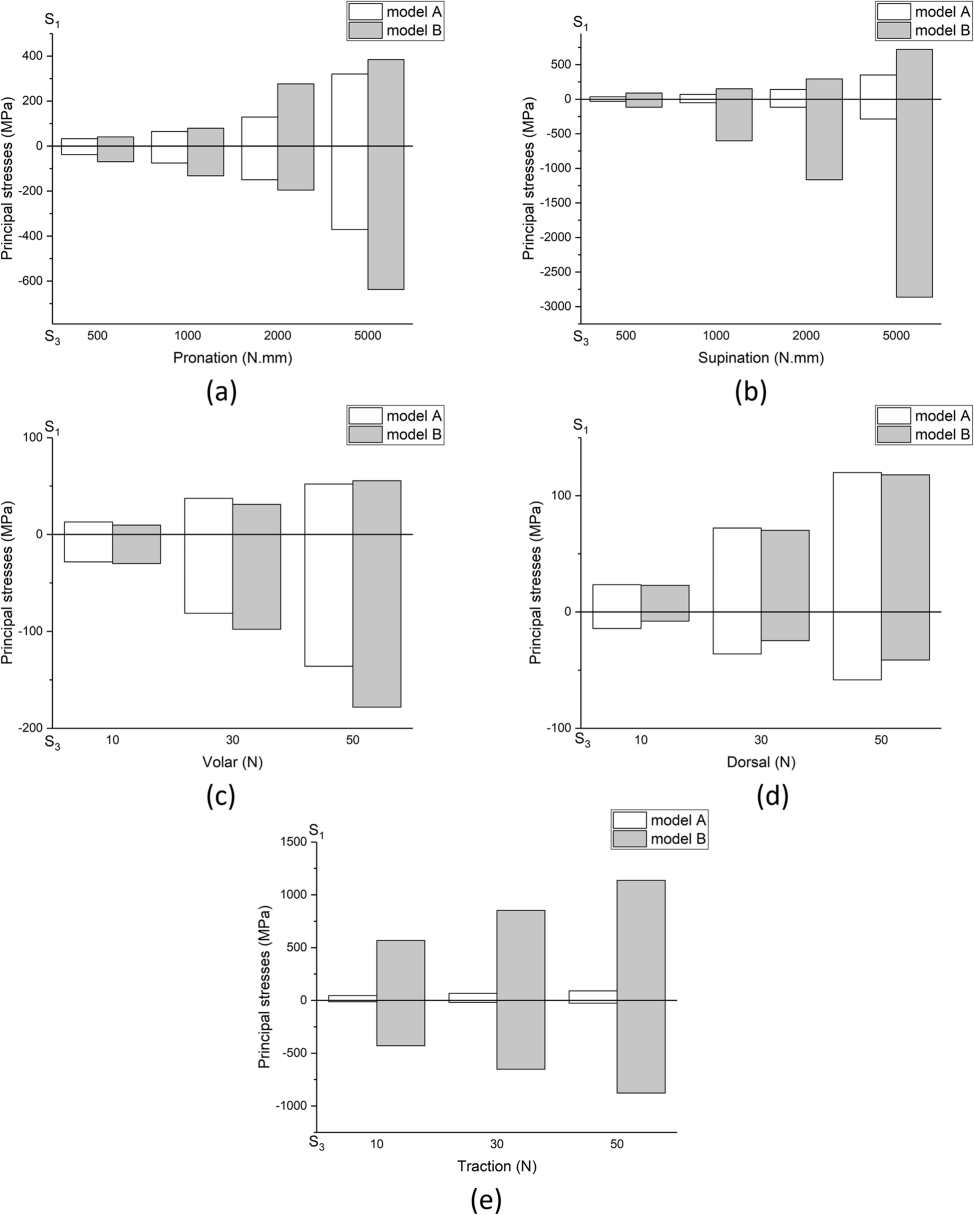
The amount of maximum and minimum principal stress in all conditions performed during movements: (**a**) pronation, (**b**) supination and loading: (**c**) volar, (**d**) dorsal, (**e**) traction

**Fig. 7:**
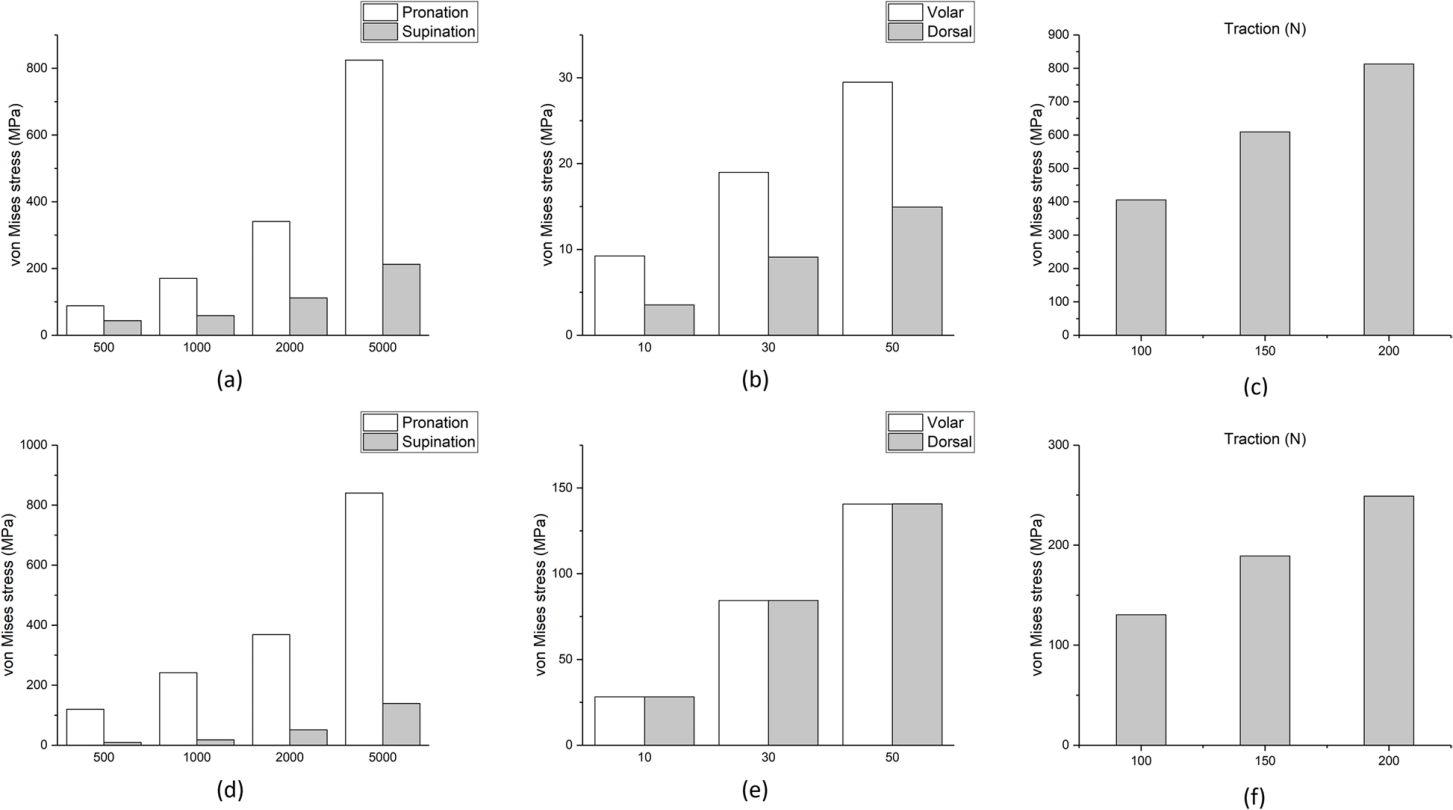
The amount of von Mises stress in all conditions performed for screws: (**a**) pronation and supination, (**b**) volar and dorsal, (**c**) traction, and prosthesis: (**d**) pronation and supination, (**e**) volar and dorsal, (**f**) traction

Figure 5 shows the distribution of von Mises stress in each loading condition in the prosthesis and the screws. As can be seen, the prostheses and screws are subjected to higher stresses during pronation than supination. Also, the amount of stress created in prostheses and screws during volar and dorsal loading is almost equal. In traction loading, screws are subjected to very high stresses. For example, the amount of stress obtained in the prosthesis and screws during the pronation and under 1000 N.mm loading was 220.13 MPa and 180.3 MPa, respectively, and the equivalent of this loading during supination was 20.5 MPa and 80.3 MPa, respectively. Furthermore, by comparing the stresses created in the prosthesis and the screw during supination and pronation, it can be observed that in supination, the screws are significantly under more stress than the prosthesis. Still, the prosthesis is slightly more stressed than the screws in pronation. The displacement in the bone and prosthesis in model B during the pronation and supination is significantly higher than in model A. In both models, no significant difference was observed during volar and dorsal loading. While in the traction loading, there is higher displacement in model B. The results obtained in all loading conditions during supination and pronation, the forces applied in volar, dorsal, and traction loading are reported in Tables 1, 2, and 3, respectively, and the comparison of these results is shown in the following diagrams (Figs. 6, and 7).

**Table 1:** Stress and displacement values in supination and pronation

Loading condition			500 (N.mm)	1000 (N.mm)	2000 (N.mm)	5000 (N.mm)
Supination	model A	Maximum principal stress (MPa)	35.00	70.30	140.8	351.9
		Minimum principal stress (MPa)	-28.88	-57.71	-115.2	-286.2
		Displacement (mm)	2.35	4.38	8.80	22.27
	model B	Maximum principal stress (MPa)	80.70	152.1	294.1	722.2
		Minimum principal stress (MPa)	-115.3	-603.0	-1166	-2863
		Displacement (mm)	7.02	14.83	28.23	65.90
	prosthesis	von Mises stress (MPa)	10.3	20.5	41.36	61.4
	screws	von Mises stress (MPa)	50.12	80.3	110.0	200.1
Pronation	model A	Maximum principal stress (MPa)	32.83	65.0	128.9	320.1
		Minimum principal stress (MPa)	-37.59	-74.92	-149.2	-370.3
		Displacement (mm)	1.47	2.66	5.20	13.10
	model B	Maximum principal stress (MPa)	40.76	79.25	276.3	384.6
		Minimum principal stress (MPa)	-68.97	-132.1	-195.2	-637.2
		Displacement (mm)	20.92	42.0	83.0	140.2
	prosthesis	von Mises stress (MPa)	110.2	220.13	380.7	800.1
	screws	von Mises stress (MPa)	90.4	180.3	360.6	800.0

**Table 2:** Stress and displacement values in volar and dorsal loads

			10(N)	30(N)	50(N)
Volar	model A	Maximum principal stress (MPa)	12.89	37.26	62.12
		Minimum principal stress (MPa)	-28.24	-81.49	-135.9
		Displacement (mm)	1.02	2.99	4.98
	model B	Maximum principal stress (MPa)	80.70	152.1	294.1
		Minimum principal stress (MPa)	-115.3	-603.0	-1166
		Displacement (mm)	0.90	2.45	4.08
	prosthesis	von Mises stress (MPa)	25.1	74.9	131.4
	screws	von Mises stress (MPa)	9.5	18.6	28.7
Dorsal	model A	Maximum principal stress (MPa)	23.42	72.18	120.0
		Minimum principal stress (MPa)	-14.01	-36.03	-58.27
		Displacement (mm)	0.89	2.71	4.52
	model B	Maximum principal stress (MPa)	22.81	70.17	118.0
		Minimum principal stress (MPa)	-8.03	-24.55	-41.18
		Displacement (mm)	1.08	2.99	4.92
	prosthesis	von Mises stress (MPa)	25.3	75.6	130.2
	screws	von Mises stress (MPa)	3.5	8.4	15.4

**Table 3:** Stress and displacement values in traction

			100(N)	150(N)	200(N)
Traction	model A	Maximum principal stress (MPa)	45.10	67.73	90.39
		Minimum principal stress (MPa)	-12.78	-19.19	-25.5
		Displacement (mm)	5.00	7.46	9.95
	model B	Maximum principal stress (MPa)	568.3	852.5	1137.0
		Minimum principal stress (MPa)	-429.3	-652.2	-876.8
		Displacement (mm)	11.94	18.12	24.27
	prosthesis	von Mises stress (MPa)	130.14	190.05	248.63
	screws	von Mises stress (MPa)	400.21	604.3	816.41

## Discussion

This study investigated the mechanical behavior of the DRUJ prosthesis introduced in the previous study [16] by applying various forces. The finite element method was used to compare the DRUJ prosthesis with a healthy forearm. This study's results can guide the use of this prosthesis in clinical surgeries and improve its design.

In this study, we examined stress distribution in bone and prostheses. As shown in Fig. 7, the stress created in the forearm bone during the pronation is significantly less than the supination. However, the stress created in the prosthesis and screws during the pronation is more than the stresses created with supination. Also, bone and prosthesis displacement during pronation was greater than supination. By comparing these results, we conclude that when the stress on bone is smaller, loosening the screws and components of the prosthesis is less likely, which can cause a reducing the instability and failure of the prosthesis. On the other hand, due to the obtained values, especially in the cases with higher loads, it is necessary to do more research on the design and placement of the prosthesis.

Some studies in forearm computational analysis have focused on the kinematic and instability of the DRUJ, and few studies have examined the biomechanical behavior of the joint and DRUJ prosthesis under various loading conditions [2]. Khuyagbaatar et al. evaluated the stability of the DRUJ when using a stabilizer [20]. Bajuri et al. investigated the stress distribution in wrist arthroplasty in rheumatoid arthritis [22]. Austman et al. compared the stresses on the bone before and after using a cemented distal ulnar implant [3]. Tan et al. simulated the Monteggia fracture using the finite element method [23]. Zhang et al. evaluated locking plates in head fracture fixation [19]. Although these studies provide valuable information in DRUJ computational analysis, none of them simulated the distal prosthesis under different loading conditions.

Few clinical studies also investigated the prosthesis behavior planted on a cadaver. A clinical study analyzed five patients (four men and one woman) with a mean age of 48.8 years and a mean follow-up of 27.6 months who experienced intraosseous DRUJ replacement surgery [15].In that study, the stability of the prosthesis during traction force loading showed significant resistance by applying traction force of 150 N, and no dislocation was observed in the prosthesis. However, in the present study, as shown in Fig. 7, the bone in model B is subjected to significant stress during this loading condition. Also, hand and prosthesis displacement with this loading is higher than the displacement in a healthy case. On the other hand, although the amount of stress created in the prosthesis is acceptable, the screws are under relatively high stress. Whether this amount of stress exposes the stability and function of the prosthesis to dislocation and failure requires further investigation.

As seen in Fig. 7, in examining the stress distribution in the bone and prosthesis volar and dorsal, the stresses on model B are significant during the volar loading. In contrast, in the dorsal loading, there is no considerable stress difference between Model A and B. Although the stress distribution in the prosthesis is approximately the same during both loadings, the stresses on screws are almost twice the stresses created with the volar loading. Also, the displacement in both loads is almost equal for both models, which is significant. While the results of the cadaver study reported that the prosthesis was very stable during volar loading, and no dislocation was observed while applying this loading. Still, the prosthesis was unstable during dorsal loading, and four dislocations out of 16 cadavers were recorded.

In this study, as in other similar studies, there are some limitations that should be considered in the future. Simulation of the wrist area was not performed due to simplification. Considering this area can be effective in the better application of boundary conditions. The applied loads are concentrated, while for more realistic results, it is better to use the loads through the reaction of the muscles [24]. The mechanical properties of ligaments and cartilages were considered linearly elastic, while ligaments and cartridges exhibit time-dependent nonlinear behavior [25]. Since bone is anisotropic, applying anisotropic properties to bone can lead to more accurate results, which should be considered in the future studies. Using a viscoelastic model for the ligament can be beneficial in achieving better results. Although, in this study, the cortical and trabecular parts of the bone were identified, the mechanical properties of the bone have a heterogeneous distribution and change based on osteoblasts and osteoclasts interaction due to the well-known bone remodeling procedure [26, 27]. Considering bone remodeling and the effects of bone damage due to overloading of the functions that the prosthesis may cause can be crucial in better studying the behavior of the prosthesis. The constructed geometry in this study was based on CT images of a 33 years old healthy man, so the assigned material properties were chosen based on data available for healthy bone. As we know, in the presence of diseases like osteoarthritis, the stability and function of the prosthesis are greatly affected. Therefore, considering patients with such diseases and assigning the related properties can open a new perspective in studying the DRUJ prosthesis. Finally, a case study on this type of prosthesis recipient and applying mechanical properties and boundary conditions according to the patient's characteristics can provide helpful information and effective future clinical decisions.

## Conclusion

This study showed that the DRUJ prosthesis could help restore the healthy hand’s functionality but shows a tendency to fail under unreal overloading conditions. However, it should be noted that initially, the screws are subjected to more stress during the pronation, and it is essential to know where the bone will form. But in supination, the most stressed part is the prosthesis, which may lead to its failure. In addition, there is a possibility of dislocation after osseointegration during the supination. Considering the failure observed in clinical practice, finite element analysis of intraosseous prostheses can effectively decrease mechanical problems. The results obtained in this study are promising, and the stresses obtained as a result of normal loading do not exceed the strength of the material. The design of the prosthesis should be optimized if it is vital to use such a prosthesis for a particular case that requires overloading.

## Data Availability

The datasets used and/or analyzed during the current study available from the corresponding author on reasonable request.

## References

[1] Fuchs N, Meier L, Giesen T, Calcagni M, Reissner L (2020). Long-term results after semiconstrained distal radioulnar joint arthroplasty: A focus on complications. Hand Surg Rehabil.

[2] Thomas BP, Sreekanth R (2012). Distal radioulnar joint injuries. Indian J Orthop.

[3] Austman RL, King GJ, Dunning CE (2011). Bone stresses before and after insertion of two commercially available distal ulnar implants using finite element analysis. J Orthop Res.

[4] Darrach W (1992). Partial Excision of Lower Shaft of Ulna for Deformity Following Colles's Fracture. Clin Ortho Related Res (1976-2007).

[5] Bieber EJ, Linscheid RL, Dobyns JH, Beckenbaugh RD (1988). Failed distal ulna resections. J Hand Sur.

[6] Field J, Majkowski R, Leslie I (1993). Poor results of Darrach's procedure after wrist injuries. Journal Bone Joint Surg Br.

[7] Minami A, Iwasaki N, Ishikawa J-i, Suenaga N, Yasuda K, Kato H (2005). Treatments of osteoarthritis of the distal radioulnar joint: long-term results of three procedures. Hand Surgery.

[8] Bowers WH (1985). Distal radioulnar joint arthroplasty: the hemiresection-interposition technique. J Hand Surg.

[9] Kapandji I (1986). The Kapandji-Sauvé operation. Its techniques and indications in non rheumatoid diseases. Ann Chir Main.

[10] Lees V, Scheker L (1997). The radiological demonstration of dynamic ulnar impingement. J Hand Surg.

[11] Hagert C-G (1992). The distal radioulnar joint in relation to the whole forearm. Clin Orthop Relat Res.

[12] Laurentin-Pérez L, Goodwin A, Babb B, Scheker L (2008). A study of functional outcomes following implantation of a total distal radioulnar joint prosthesis. J Hand Surg (Eur Volume).

[13] Moradi A, Binava R, Vahedi E, Ebrahimzadeh MH, Jesse BJ (2021). Distal Radioulnar Joint Prosthesis. Arch Bone Joint Surg.

[14] van Schoonhoven J, Fernandez DL, Bowers WH, Herbert TJ (2000). Salvage of failed resection arthroplasties of the distal radioulnar joint using a new ulnar head prosthesis. Journal Hand Surg.

[15] Moradi A, Binava R, Hasanabadi SE, Vahedi E, Ebrahimzadeh MH (2020). Introduction and Early Outcomes of Intraosseous Distal Radioulnar Joint Prosthesis: A Pilot Study and a Technique on a New Design of the Sauvé-Kapandji Procedure. Arch Bone Joint Surg.

[16] Moradi A, Binava R, Hedjazi A, Hasanabadi SE, Chaharjouy NT, Ebrahimzadeh MH (2021). Biomechanical Evaluation of Intraosseous Distal Radioulnar Joint Prosthesis: A Prosthesis Designed Based on Sauvé-Kapandji Procedure. Orthop Traumatol Surg Res.

[17] Miguel-Andres I, Alonso-Rasgado T, Walmsley A, Watts AC (2017). Effect of anconeus muscle blocking on elbow kinematics: electromyographic, inertial sensors and finite element study. Ann Biomed Eng.

[18] Rezende CEE, Chase-Diaz M, Costa MD, Albarracin ML, Paschoeto G, Sousa EAC, Rubo JH, Borges AFS (2015). Stress distribution in single dental implant system: three-dimensional finite element analysis based on an in vitro experimental model. J Craniofacial Surg.

[19] Zhang Y, Shao Q, Yang C, Ai C, Zhou D, Yu Y, Sun G (2021). Finite element analysis of different locking plate fixation methods for the treatment of ulnar head fracture. J Orthop Surg Res.

[20] Khuyagbaatar B, Lee S-J, Cheon M, Batbayar T, Ganbat D, Kim YH. Effect of wrist-wearing distal radioulnar joint stabilizer on distal radioulnar joint instability using a forearm finite element model. J Mechanical Sci Technol. 2019;33(5):2503–8.

[21] Dhason R, Roy S, Datta S (2020). A biomechanical study on the laminate stacking sequence in composite bone plates for vancouver femur B1 fracture fixation. Comput Methods Programs Biomed.

[22] Bajuri M, Kadir MRA, Murali MR, Kamarul T (2013). Biomechanical analysis of the wrist arthroplasty in rheumatoid arthritis: a finite element analysis. Med Biol Eng Compu.

[23] Tan J, Mu M, Liao G, Zhao Y, Li J (2015). Biomechanical analysis of the annular ligament in Monteggia fractures using finite element models. J Orthop Surg Res.

[24] Ashrafi M, Ghalichi F, Mirzakouchaki B, Doblare M (2021). On the effect of antiresorptive drugs on the bone remodeling of the mandible after dental implantation: a mathematical model. Sci Rep.

[25] Ashrafi M, Ghalichi F, Mirzakouchaki B, ZoljanahiOskui I (2020). Numerical simulation of hydro-mechanical coupling of periodontal ligament. Proc Inst Mech Eng [H].

[26] Ashrafi M, Gubaua JE, Pereira JT, Gahlichi F, Doblaré M (2020). A mechano-chemo-biological model for bone remodeling with a new mechano-chemo-transduction approach. Biomech Model Mechanobiol.

[27] Ashrafi M, Ghalichi F, Mirzakouchaki B, Arruga A, Doblare M (2020). Finite element comparison of the effect of absorbers' design in the surrounding bone of dental implants. Int J Numer Methods Biomed Eng.

